# Thoughts Relative to Artificial Dentures*Read before the Northern Illinois Dental Society, October, 1912, and published in the May Dental Review.

**Published:** 1913-05-15

**Authors:** Irvin B. Carolus

**Affiliations:** Sterling, Ills.


### THOUGHTS RELATIVE TO ARTIFICIAL DENTURES.*

BY DR. IRVIN B. CAROLUS, STERLING, ILLS.

There never was a time probably in the history of the profession when the demands were so great and the needs so limited for this class of work. Public ignorance, inconsiderate, indifference, and avaricious commercialism on the part of many practitioners, form the foundation upon which is built the pernicious standards that stare in our faces unremittingly. With it some build practices, build homes and fortunes. Some build curses and disrepute. Whatever else they may be doing for self or patient, they are producing an hereditary maxillary deformity and psychological condition, which will require several generations, and perhaps many more, for Nature, with our persistent assistance to rectify. As proof of this contention I ask you to be serious, and direct your attention to the contracted impacted-molar jaw, the irregular dental arch with its accompanying terrible afflictions including practically all the diseases of the oral cavity with those of the nose and throat, the whole alimentary tract, and in their turn, all of the organs of vitality crying out for proper mastication, digestion and assimilation.

Fellow workers, here is a supreme responsibility! Why do we pass it so lightly, and why are there not many more to carry out this work of prevention instead of cure? To promote this interest we must be teachers. No one else will educate the public. Our efforts should be directed towards the spirit of the times—aim to save, not to destroy—not be the first to break the permanent dental arch; nor that of the deciduous, before the proper time. If extraction is persistently insisted upon, send it away and shift

*Read before the Northern Illinois Dental Society, October, 1912, and published in the May DENTAL REVIEW.

the responsibility to someone else who is cowardly enough to do it. We cannot afford to do a wrong to others which we would not tolerate for ourselves.

We do, however, occasionally find neglected cases, for which extraction and artificial dentures is the inevitable result. Here then it behooves us to put forward our best efforts—the very best that we possess—producing an artificial substitute for the failure that Nature intended well.

Being positive of the diagnosis it is much better to treat such cases preparatory to an appointed time for the surgical part of the work, which should require of us such care and attention to perfect cleanliness, neat operating in extraction, replacing or removing distorted or prominent portions of the process, dressing lesions, etc., that it will command a compensating fee of itself—not a deposit that ties a string to the plate. The writer has never been able to convince himself of this charitable (?) tendency of so many Doctors of Dental Surgery who give to the laity free of charge such an important corner of the sheepskin. Little things like this and many others, tend to measure us in public opinion. But public opinion should not be our whole ambition. We should do it because it is right; because it is justice to self and justice to others. We are professional men and need a better opinion of ourselves and our services.

Each and every one of you can take an impression and make a plate more quickly than this paper could tell it, and perhaps better. The technic you have learned well and practiced so often, that general ideas cannot interest you. Individuality and special experience calls us together for exchange of ideas and methods for better service, so let us consider a few points of greater or less importance in detail of rubber dentures; though they may be applied to all others as well.

Every dental laboratory should have a liberal and well-selected assortment of impression trays. When new they should be given a coat of thin solution wax and gasoline. After being used they should be boiled, wiped dry and coated again. This prevents discoloration, and the ease with which tray and impression can be separated by simply holding over heat for a moment will amply repay for the effort to keep the tray like new and always ready.

Plaster of Paris is the standard impression material perhaps the best though there are cases, and especially of the lower, that better results can be obtained by using fresh low-heat modelling compound. Use as little impression material as you can consistently. There is great danger to the results in using more than is absolutely necessary.

In taking an upper impression patient should sit erect; tray inserted, pressed firmly to place and held with one finger on center of palate; then incline patient very much forward to the point of gravitation. This position you will find very beneficial in relaxing muscles, and preventing nausea. Do not remove until chemical action has made the tray very warm. A good story will help to pass the time, and if you gauge it correctly, the climax will come just at the right time and you will find the hearty laugh will assist in removing the tray more easily than a forced cough.

The bite taken with modelling compound in an upper and lower bite-tray will be very efficient though not final for it is not wholly dependable and requires the trial wax plates for assurance of accuracy. Place the tray in the patient's mouth, instruct to close just enough to retain it; then with one hand on the back of the neck and the other on the forehead, tilt the head backward until the muscles of the throat are tense; then require the closing slowly to the point of proper proximity of upper and lower, and mark the mesial and bite line. This method rarely fails to give the correct rest bite, and you will find its accuracy is more certain and the simplicity of manupilation is much ahead of other means and devices you may have tried.

Varnish the plaster impression with shellac. When dry, dust with French chalk and rub it smooth with the finger. This will give a splendid surface for the model and make easy separation. Pour model and let set for several hours, preferably over night, which can easily be arranged by making the appointment toward the close of the day. When the model and impression are separated, mark the border line of rims and palate, cutting away the plaster to the depth of carboard thickness graduating to nothing at the edge of the ridge, except at the palatine border where it may be graduated anteriorly for about an eighth of an inch. For this portion the groove may be deepened slightly at the softer parts of the palate between the median line and the condyles. All nicely trimmed it is now ready for more French chalk, making a fine smooth dense surface enabling the easy removal of the wax trial, and will also present a splendid surface for vulcanization. Place models in bite, and set in the articulator. Be positive of the median, horizontal and vertical lines; also, the relation of the bite-line to and distance of models from the hinge of the articulator. In other words, the articulated models must represent the anatomical parts. Diligent care in this manipulation eliminates the possibilty of future grinding of the porcelain.

To differentiate between the steps required in the production of an artificial case, and point to one part as more important than another, would meet with a variance of opinions. While all are exceedingly important, this work we have to do with the articulator requires a considerably higher development of artictic qualifications to produce esthetic results, thereby approaching most nearly the natural in appearance and function. It is very simple to employ mechanical methods and make a set of teeth; and how often we see the results of such carelessness—so hideously artificial that we mutely censure the fellow who did it. What we really need and desire is art which conceals art. Then let us not be content to cease our efforts until we have attained that condition—a denture which will give to the patient that conformity of facial feature; that harmony of mould; that exquisite blend of shade; the proper relation of bite-line to, and sufficient room for that important assistant to mastication, the tongue; the correct overbite which plays its wonderful part in speech; and the grinding or macerating surfaces for lateral manipulation as well as the chopping. All together restoring appearance and utility as nearly as we are able to cope with nature. The wax trial plate with teeth set up, gums nicely carved, and results of absorption restored—just as we wish the finished denture, is the working model we have to obtain the result; and it may be necessary to try and try again until it is just right. Each individual case requires its own special care and consideration. No two are just alike any more than the two individuals.

Tooth manufacturers have succeeded well in giving us shades, but there is a great opportunity for improvement in the production of better moulds—something more nearly imitating the natural organs so that it will not be necessary to spend so much time in reshaping to suit the requirements. But it is time well spent. The work is fascinating and the result well pays for the effort. It leads us in new fields and broadens our knowledge of human nature, in the study of temperament and character.

Did you ever notice the individuality of the dental office displayed in an artificial denture—how accurately and unintentionally certain mechanical effects produced are recognized as that coming from some brother's earnest application to duty? Whether you have or have not, it still remains a fact. This individuality is the design of the Creator, and is a part of our being. We cannot overcome it if we would; and would it be wise if we could? It serves well as an illustration of what we should attempt to do—to produce an artificial substitute so natural and becoming that we might as easily be able to recognize the father or the mother of the patient through family character lines displayed in the teeth as we do the maker of the artifice.

Experience has taught that with good methods and careful manipulation, the old fashioned horseshoe-shaped suction chamber and the newer-idea suction cup are decidedly unnecessary. But as a precaution to rapid alveolar absorption and slight assistance to the inexperienced patient it is well to make what might be called a relief extending over the whole palate of the plate and overlapping the palatine border of the alveolar ridge. This is accomplished by burnishing one thickness of tea-lead on the model after the flask has been cleaned ready for packing. A double thickness covering the hard portion of the palate along the median line is advisable in a case of that character. The impression should not be scraped. The rugae should be saved and carried to the lingual surface of the plate as prominently as possible.

The finishing of the plate is an important factor many times hurriedly overlooked. All rims and borders should have rounded polished surfaces—the palatine extremity graduated to a nice thin margin. The mucous surfaces of the plate should be polished as nicely as the labial and lingual. Its natural undulations must be preserved with that smoothness of surface which renders it non-irritating and easily cleaned.

We have now come to the point of greatest anxiety, perhaps, and especially to the younger practitioner—the appointed time for the handiwork of man and the handiwork of God to be joined together. What a moment of suspense as the newly made plate is being placed in position! What joy or what gloom! Will the wings droop beatenly, or will the chest swell up in proud distinction? What is the use? Why not overcome such uncalled-for state of mind and be passively natural? It is too early to exult with joy, your duty is not ended thus far, you are awaiting an early subsequent appointment and more if necessary until such time as the conditions prove the efforts have been the success intended. In this way it is easy to overcome that humiliating moment every one of us has experienced in hearing, "Well, now I'll try it to see if it is alright." It will be alright if you make it so, and think it, and impress the patient with your position. On the other hand if Fate seems to have worked against you and the thing does not stick or jibe just right, it is too late to feel badly, you should have rectified that error at its proper time. Perhaps it was the impression, the bite, the selection of shade, the mould, the articulation, trimming the model, the packing, the flask-closing, the vulcanization, the cooling, the finishing, or perhaps it was the plaster-mix. Whichever it was you knew it at the time or should have known it. The trouble was due to carelessness and it is a very easy matter to prove it. "Be sure you are right, then go ahead." It may take a little more time than to do it hurriedly; it demands more concentration of thought, but it pays to be positive and know just exactly what the finished work will be. Get better fees and give the people better service. If you don't some competitor will. Let others say, "He is a high-priced man but a mighty fine dentist." I can't conceive of a nicer compliment, can you?

To be sure, the road to such success seems rough and almost impossible; but those who are not naturally endowed with attractive personality must and do make up for it in closer application to those qualities which may be acquired; so that on final standard measurement we are largely on a par, and it is within our pleasure to so conduct our professional career that we may feel the sense of gratification that we have done our duty well.

Each new achievement leads to grander success and higher rewards; elevates our professional standing, increases our own confidence, and serves humanity—our greatest ambition.

